# Acute ischemic stroke in Tsutsugamushi: understanding the underlying mechanisms and risk factors

**DOI:** 10.1186/s12883-024-03534-1

**Published:** 2024-01-24

**Authors:** Dain Kim, Yeon Hee Cho, Jeong Bin Bong, Young Seo Kim, Byoung-Soo Shin, Hyun Goo Kang

**Affiliations:** 1 Medical School, Jeonbuk National University, Jeonju, 54907 South Korea; 2 Department of Neurology, Chosun University School of Medicine, Gwangju, 64153 South Korea; 3 Department of Neurology, Wonkwang University School of Medicine, Iksan, 54538 South Korea; 4 Department of Neurology, Research Institute of Clinical Medicine of Jeonbuk National University, 20 Geonji-ro, Deokjin-gu, Jeonju-si, 54907 Jeonbuk-do South Korea; 5 Biomedical Research Institute, Jeonbuk National University Medical School and Hospital, 20 Geonji-ro, Deokjin-gu, Jeonju, 54907 Jeonju South Korea

**Keywords:** Stroke, Ischemic stroke, Scrub typhus, Infection

## Abstract

**Background:**

Tsutsugamushi (scrub typhus) is an acute infectious febrile disease common in the Asia-Pacific region. Common symptoms of tsutsugamushi include lymphadenopathy, fever, and myalgia, and it rarely causes acute ischemic stroke (AIS). However, we hypothesized that tsutsugamushi infection could trigger AIS.

**Method:**

We retrospectively examined patients diagnosed with AIS within 2 weeks of tsutsugamushi diagnosis at three hospitals over a 15-year period. We categorized patients who developed AIS while being treated for tsutsugamushi as the case group and those (of similar age and sex) who did not develop AIS as the control group. The case and control groups consisted of 22 and 66 participants, respectively. When a scattered pattern was observed or lesions were found in two or more vascular territories on diffusion-weighted imaging, the pattern was defined as embolic. Other patterns were defined as nonembolic.

**Results:**

Among the 19 patients, excluding three with transient ischemic stroke, 15 (78.9%) showed an embolic pattern. Although fever was common in the control group, it was less common in the case group. A higher D-dimer level at the time of hospitalization was associated with the development of AIS in patients with tsutsugamushi.

**Conclusions:**

AIS in patients with tsutsugamushi showed an embolic rather than a non-embolic pattern on brain magnetic resonance imaging. It was more likely to occur in patients with risk factors for stroke. Tsutsugamushi patients with AIS were likely to have no fever or high D-dimer levels. We hypothesized that D-dimers play an important role in the pathophysiology, where tsutsugamushi infection increases the likelihood of AIS.

**Supplementary Information:**

The online version contains supplementary material available at 10.1186/s12883-024-03534-1.

## Background

Tsutsugamushi, also known as scrub typhus, is an acute infectious febrile disease caused by the bacterium *Orientia tsutsugamushi*; it is prevalent in Asia and the islands of the Pacific and Indian Oceans [1]. In particular, in 2022, 6,232 patients with tsutsugamushi were reported in South Korea, with approximately 5,000 cases per year in the last 5 years, thus showing a high incidence [2].

Common symptoms of tsutsugamushi include eschar, lymphadenopathy, fever, headache, myalgia, and gastrointestinal symptoms; severe symptoms of the condition include interstitial pneumonia, acute respiratory distress syndrome, meningoencephalitis, acute kidney injury, and disseminated intravascular coagulation (DIC) [1]. Although acute ischemic stroke (AIS) is a rare symptom of tsutsugamushi, some patients present with both tsutsugamushi and AIS in clinical practice. Similar cases have been reported infrequently. We hypothesized that tsutsugamushi infection could be a causal factor of AIS because acute infections, of which tsutsugamushi infection is a type, could increase the incidence of AIS [3]. This study aimed to investigate the specific mechanisms underlying the development of AIS in patients with tsutsugamushi.

The main pathophysiology of tsutsugamushi affecting the blood vessels comprises endothelial damage and vasculitis. However, because AIS is rare in patients with tsutsugamushi infections, we suspected that there may be mechanisms other than vasculitis. This study hypothesized that tsutsugamushi-induced coagulation disorder leads to AIS based on the results of previous studies, which suggested that tsutsugamushi may cause a disorder in the coagulation pathway [4] and that thrombogenesis is the infection’s main mechanism underlying stroke induction [5]. To test this hypothesis, we analyzed the data of patients with tsutsugamushi and AIS.

## Methods

### Patient selection

This study retrospectively evaluated patients who were hospitalized for tsutsugamushi at three regional hub hospitals in different urban areas over a 15-year period (2005–2019) and were diagnosed with AIS within 2 weeks of tsutsugamushi diagnosis. We collated and analyzed the symptoms, medical histories, and laboratory findings of the patients at the time of admission using electronic medical records. We categorized patients who developed AIS while being hospitalized and treated for tsutsugamushi as the case group; those who did not develop AIS while being hospitalized and treated for tsutsugamushi at the same hospital and were similar in age and sex to the case group were categorized as the control group. As a result, this study categorized 22 and 66 patients into the case and control groups, respectively. This study was conducted in accordance with the Declaration of Helsinki and was approved by the Institutional Review Board of Jeonbuk National University Hospital (CUH 2023-05-001). The requirement for informed consent was waived owing to the retrospective nature of the study.

### Definition

Patients were diagnosed with tsutsugamushi infection when the *O. tsutsugamushi* gene was detected by polymerase chain reaction (PCR), *O. tsutsugamushi* was identified in the serum, or IgM antibodies against *O. tsutsugamushi* were detected. The AIS group included patients with acute ischemic stroke confirmed using brain magnetic resonance imaging (MRI) and those with transient ischemic stroke (TIA). However, this study excluded patients with subacute or chronic stroke. TIA was defined as the presence of stroke-related neurological symptoms, such as dysarthria or hemiplegia, that improved within 24 h. Patient symptoms were identified based on the presenting symptoms at the time of hospitalization. Furthermore, the present study examined basic demographic information (age and sex), presence of eschar due to tsutsugamushi, and various tsutsugamushi-related symptoms (fever, headache, fatigue, anorexia, dyspepsia, nausea, vomiting, abdominal pain, diarrhea, arthralgia, myalgia, sore throat, back pain, dyspnea, cough, and thirst). In addition, we recorded the antibiotics used for tsutsugamushi treatment, measured the time from admission to the development of AIS symptoms, and investigated the risk factors for stroke (hypertension, diabetes mellitus, atrial fibrillation, dyslipidemia, coronary arterial disease, drinking history, and smoking history) at the time of admission. Regarding stroke risk factors, a patient was defined as having a risk factor as an underlying disease if the patient had been diagnosed with a risk factor and was receiving medication for it or if the patient met the relevant diagnostic criteria after hospitalization, even though the patient was not diagnosed with a risk factor. Hypertension was defined as a state in which blood pressure measured at rest during hospitalization was ˃130/80 mmHg [6]. Diabetes mellitus was diagnosed if the 8-h fasting blood glucose level was 126 mg/dL or higher, HbA1c was 6.5% or higher, or blood glucose was 200 mg/dL or higher 2 h after the oral glucose tolerance test [7]. Atrial fibrillation group included all patients who had atrial fibrillation confirmed by electrocardiography performed after admission or those previously diagnosed with atrial fibrillation. Dyslipidemia was diagnosed if the low-density lipoprotein level was 160 mg/dL or higher, triglyceride level was 200 mg/dL or higher, high-density lipoprotein level was 40 mg/dL or lower, or total cholesterol level was 240 mg/dL or higher [8–10]. Coronary artery disease was diagnosed if abnormal findings were confirmed on coronary angiography. If a patient’s response showed that the patient had consumed alcohol or cigarettes within 1 year of hospitalization, the patient was defined as having a drinking or smoking risk factor. Aspartate aminotransferase (AST), alanine aminotransferase (ALT), white blood cell (WBC), hemoglobin (Hg), and platelet (PLT), laboratory findings that generally show abnormal values in tsutsugamushi patients [11], and prothrombin time (PT), activated partial thromboplastin time (aPTT), D-dimer, fibrinogen, and fibrin degradation product (FDP), which are blood coagulation-related laboratory findings that may affect ischemic stroke, were investigated using blood tests performed during hospitalization.

### Imaging analysis

This study investigated the pattern of lesions caused by stroke by collecting and analyzing brain MRI scans of patients admitted for tsutsugamushi and diagnosed with acute ischemic stroke or TIA during treatment. Subsequently, we examined the presence of atrial fibrillation and D-dimer levels, which are believed to be associated with embolic patterns. High D-dimer levels and embolic patterns are related because high D-dimer levels means hyper-coagulable state, which in turn causes the generation of thromboembolism. Since atrial fibrillation is one cause of embolic stroke, patients who already suffer from atrial fibrillation are likely to have developed embolic stroke due to atrial fibrillation regardless of the presence or absence of tsutsugamushi infection. Therefore when analyzing the MRI patterns of tsutsugamushi patients with AIS, the history of atrial fibrillation must be referred to. When a scattered pattern (indicating a high signal intensity scattered over multiple territories) was observed on diffusion-weighted imaging (DWI) or when lesions were present in two or more different vascular territories, the pattern was defined as embolic. Other types were defined as nonembolic.

### Statistical analysis

First, a control group was selected based on age and sex matching of patients with a disposition similar to that of the case group. Age and sex matching were performed using propensity score matching and the nearest neighbor matching algorithm, and 66 patients were selected as the control group using a 1:3 matching ratio. We then compared the demographics, clinical symptoms, and stroke risk factors of patients with tsutsugamushi infection with or without AIS. Pearson’s chi-squared or Fisher’s exact tests were used to analyze categorical variables and the Student’s t-test was used for continuous variables. Continuous variables were summarized as means with standard deviations or medians with interquartile ranges, whereas categorical data were expressed as counts and percentages. Multivariate analysis was performed to identify the independent factors associated with tsutsugamushi presenting with AIS. To avoid variable selection caused by spurious correlations, only variables showing a potential association (*p* < 0.1) in univariate analysis were included as potential factors associated with tsutsugamushi presenting with AIS in the multivariate logistic regression model. Statistical significance was set at *p* < 0.05 (two-tailed). All statistical analyses were performed using SPSS 21.0 (IBM Corp., Armonk, NY, USA).

## Results

Among the 22 tsutsugamushi patients who developed AIS, 14 were females (63.6%) and eight were males (36.4%); mean age was 74.4 ± 13.4 years, with the youngest patient being 26 years old and the oldest 93 years old (Table 1). The antibiotics used for the treatment of tsutsugamushi included doxycycline in 16 patients (72.7%), azithromycin in four patients (18.2%), and rifampicin in two patients (9.1%). The average time from the onset of tsutsugamushi symptoms to that of AIS was 3.91 ± 4.54 days; meanwhile, the time of onset in four patients was ˃10 days. In addition, factors associated with vascular risk were current alcohol consumption in two (9.1%) participants, current smoking status in two (9.1%), hypertension in 12 (54.5%), dyslipidemia in seven (31.8%), diabetes mellitus in five (22.7%), atrial fibrillation in five (22.7%), and coronary artery disease in two (9.1%).

**Table 1:** Baseline characteristics of patients with tsutsugamushi and acute ischemic stroke

	Sex	Antibiotics	Symptom onset to stroke (day)	Alc	Smk	HTN	DM	A.fib	DL	CAD
**1**	F	Dox	14	0	0	0	0	0	0	0
**2**	F	Dox	1	0	0	1	1	1	1	0
**3**	M	Rip	1	0	1	0	0	0	0	0
**4**	M	Rip	9	0	0	1	1	0	1	1
**5**	M	Dox	1	0	1	0	0	0	0	0
**6**	F	Dox	1	0	0	0	0	1	0	0
**7**	F	Dox	1	0	0	1	0	0	1	0
**8**	F	Dox	1	0	0	1	1	0	1	0
**9**	M	Azi	1	0	0	1	0	1	0	1
**10**	F	Dox	11	0	0	0	0	0	0	0
**11**	F	Dox	10	0	0	1	0	0	1	0
**12**	M	Dox	1	0	0	1	0	0	1	0
**13**	F	Dox	5	0	0	1	0	0	0	0
**14**	F	Dox	13	0	0	0	0	0	0	0
**15**	M	Dox	1	0	0	0	0	0	0	0
**16**	F	Dox	3	0	0	1	1	0	1	0
**17**	F	Dox	1	0	0	1	0	1	0	0
**18**	M	Dox	0	1	0	0	1	0	0	0
**19**	M	Azi	7	1	0	0	0	0	0	0
**20**	F	Azi	1	0	0	1	0	1	0	0
**21**	F	Dox	0	0	0	1	0	0	0	0
**22**	F	Azi	3	0	0	0	0	0	0	0

F, female; M, male; Dox, doxycycline; Rip, rifampicin; Azi, azithromycin; Alc, alcohol; Smk, smoking; HTN, hypertension; DM, diabetes mellitus; A.fib, atrial fibrillation; DL, dyslipidemia; CAD, coronary artery disease

This study described the brain MRI characteristics of 19 patients with tsutsugamushi and AIS, excluding three patients with TIA in whom no lesions were observed on DWI (Supplementary table). Among the 19 patients, 15 (78.9%) showed an embolic pattern and four (21.1%) showed a non-embolic pattern, indicating that an embolic pattern was more common. Sixteen (84.2%) patients had D-dimer levels above the normal level (0.5 mg/L) and six (31.6%) patients had atrial fibrillation as an underlying condition. Among the 16 patients with elevated D-dimer levels, 13 (81.3%) had an embolic pattern and five (31.3%) had atrial fibrillation. Among the six patients with atrial fibrillation, five had elevated D-dimer levels and one did not; all six patients showed an embolic pattern. None of the 19 patients had a history of cancer.

**Fig. 1:**
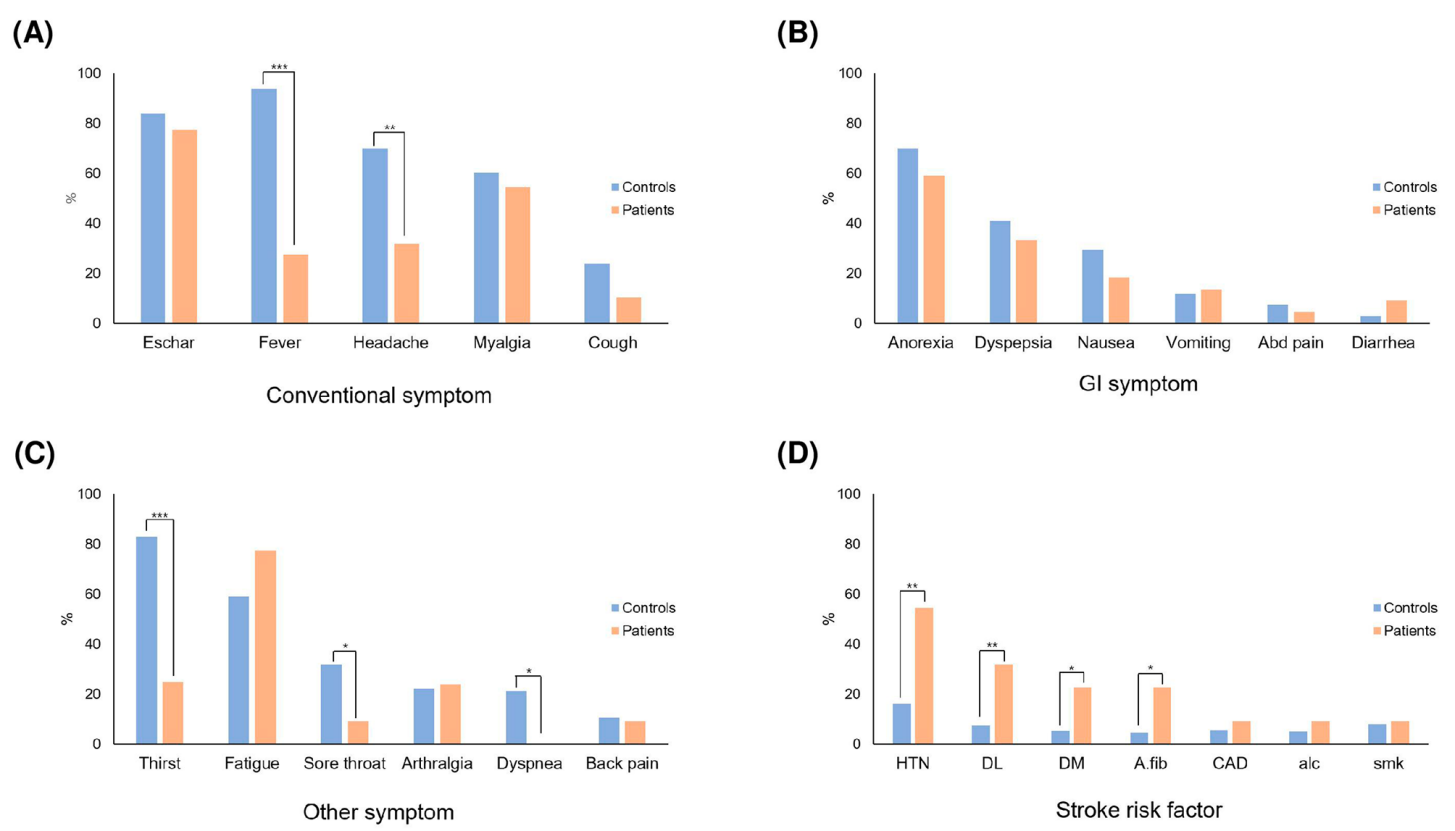
Comparison of symptoms and stroke risk factors between controls (tsutsugamushi without stroke) and patients (tsutsugamushi with stroke). Conventional symptoms of tsutsugamushi with and without acute ischemic stroke (**A**). Gastrointestinal symptoms of tsutsugamushi with and without acute ischemic stroke (**B**). Other symptoms of tsutsugamushi with and without acute ischemic stroke (**C**). Stroke risk factors in controls and patients (**D**). A statistically significant variant is expressed as *, ** or ***. * means p-value < 0.05, and ** means p-value < 0.01, and *** means p-value < 0.001

This study compared patients with tsutsugamushi infection, with and without AIS (Table 2). The mean age of the target group was 74.41 ± 13.36 years, compared with 74.18 ± 12.81 years of the control group. At the time of admission, fever was less commonly observed in tsutsugamushi patients with AIS but was significantly common in those without AIS (Tsutsugamushi patients with AIS, 27.3 vs. Tsutsugamushi patients without AIS, 93.8%, respectively; *p* < 0.001). Headache was significantly less common in tsutsugamushi patients with AIS than in those without AIS (31.8 vs. 69.8%; *p* = 0.002). The incidences of sore throat (9.1 vs. 31.8%; *p* = 0.036), dyspnea (0 vs. 21.2%; *p* = 0.018), and thirst (25.0 vs. 82.9%; *p* < 0.001) were lower in tsutsugamushi patients with AIS than in those without AIS. Tsutsugamushi patients with AIS were more likely to have stroke risk factors than those without AIS. Particularly, tsutsugamushi patients with AIS were significantly more likely to have hypertension (54.5 vs. 16.1%; *p* = 0.001), diabetes mellitus (22.7 vs. 5.3%; *p* = 0.021), atrial fibrillation (22.7 vs. 4.5%; *p* = 0.01), and dyslipidemia (31.8 vs. 7.6%; *p* = 0.004) than tsutsugamushi patients without AIS. Regarding laboratory findings, tsutsugamushi patients with AIS showed significantly higher aPTT (40.54 ± 13.47 vs. 32.31 ± 12.27; *p* = 0.009), D-dimer (8.14 ± 10.47 vs. 0.87 ± 1.27; *p* = 0.005), and FDP (42.01 ± 59.55 vs. 2.27 ± 0.69; *p* = 0.009) levels than did tsutsugamushi patients without AIS. The results in Table 2 are presented as a bar graph (Fig. 1).

**Table 2:** Comparison of tsutsugamushi patients with and without acute ischemic stroke

	Tsutsugamushi patients without stroke (*n* = 66)	Tsutsugamushi patients with stroke (*n* = 22)	P-value
**Female**	39 (59.1)	14 (63.6)	*0.706*
**Age**	74.18 ± 12.81	74.41 ± 13.36	*0.943*
**Eschar**	52 (83.9)	17 (77.3)	*0.524*
**Symptoms**			
Fever	61 (93.8)	6 (27.3)	*< 0.001*
Headache	44 (69.8)	7 (31.8)	*0.002*
Fatigue	39 (59.1)	17 (77.3)	*0.125*
Anorexia	46 (69.7)	13 (59.1)	*0.359*
Dyspepsia	16 (41.0)	7 (33.3)	*0.559*
Nausea	15 (29.4)	4 (18.2)	*0.316*
Vomiting	6 (11.8)	3 (13.6)	*1*
Abdominal pain	5 (7.6)	1 (4.5)	*1*
Diarrhea	2 (3.0)	2 (9.1)	*0.259*
Arthralgia	14 (22.2)	5 (23.8)	*1*
Myalgia	38 (60.3)	12 (54.5)	*0.636*
Sore throat	21 (31.8)	2 (9.1)	*0.036*
Back pain	7 (10.6)	2 (9.1)	*1*
Dyspnea	14 (21.2)	0 (0.0)	*0.018*
Cough	15 (23.8)	2 (10.5)	*0.335*
Thirst	29 (82.9)	4 (25.0)	*< 0.001*
**Stroke risk factors**			
Hypertension	9 (16.1)	12 (54.5)	*0.001*
Diabetes mellitus	3 (5.3)	5 (22.7)	*0.021*
Atrial fibrillation	3 (4.5)	5 (22.7)	*0.01*
Dyslipidemia	5 (7.6)	7 (31.8)	*0.004*
Coronary artery disease	3 (5.5)	2 (9.1)	*0.612*
Alcohol	2 (5.1)	2 (9.1)	*0.615*
Smoking	3 (7.9)	2 (9.1)	*1*
**Laboratory findings**			
PT	12.47 ± 1.06	14.18 ± 7.53	*0.302*
aPTT	32.31 ± 12.27	40.54 ± 13.47	*0.009*
D-dimer	0.87 ± 1.27	8.14 ± 10.47	*0.005*
Fibrinogen	292.96 ± 98.15	252.62 ± 106.69	*0.132*
FDP	2.27 ± 0.69	42.01 ± 59.55	*0.009*
AST	194.18 ± 860.00	99.82 ± 113.20	*0.611*
ALT	140.67 ± 629.12	58.95 ± 55.11	*0.546*
WBC	8.37 ± 3.48	9.54 ± 4.62	*0.213*
Hg	12.23 ± 1.85	11.49 ± 2.02	*0.117*
PLT	170.40 ± 94.30	176.55 ± 165.69	*0.831*
Creatinine	1.28 ± 0.71	1.44 ± 0.67	*0.373*

PT, prothrombin time; aPTT, activated partial thromboplastin time; FDP, fibrinogen degradation product; AST, aspartate aminotransferase; ALT, alanine aminotransferase; WBC, white blood cell; Hg, hemoglobin; PLT, platelet

Multivariate analysis was performed to identify factors associated with the development of AIS in patients with tsutsugamushi (Table 3). Known risk factors for stroke, including hypertension, diabetes mellitus, atrial fibrillation, and dyslipidemia did not show significant results. However, higher D-dimer level at the time of hospitalization owing to tsutsugamushi was found to affect the development of AIS in tsutsugamushi patients (adjusted odds ratio [OR], 1.58; 95% confidence interval [CI], 1.06–2.34; *p* = 0.023).

**Table 3:** Multivariate analysis of factors associated with tsutsugamushi with acute ischemic stroke

	Crude OR (95% CI)	P-value	Adjusted OR (95% CI)	P-value
**Hypertension**	6.27 (2.08–18.85)	*0.001*	2.64 (0.24–29.65)	*0.432*
**DM**	5.29 (1.15–24.49)	*0.033*	5.86 (0.53–64.50)	*0.149*
**A.fib**	5.29 (1.14–24.48)	*0.033*	4.55 (0.61–33.75)	*0.139*
**Dyslipidemia**	4.48 (1.24–16.21)	*0.022*	0.95 (0.05–17.94)	*0.971*
**D-dimer**	1.74 (1.22–2.47)	*0.002*	1.58 (1.06–2.34)	*0.023*

OR, odds ratio; DM, diabetes mellitus; A.fib, atrial fibrillation

## Discussion

This retrospective study aimed to determine the effects of tsutsugamushi infection on AIS onset. The co-occurrence of tsutsugamushi and AIS is rare, and only a few case reports have reported their co-occurrence [12, 13]. This study collected data from 22 tsutsugamushi patients with AIS from three hub university hospitals over a 15-year period, compared them with the data of 66 tsutsugamushi patients without AIS (controls), and identified the relationship between tsutsugamushi infection and the development of AIS.

Among the 22 patients who developed AIS after tsutsugamushi infection, four patients developed AIS more than 10 days after the onset of tsutsugamushi symptoms (Table 1). The diagnosis of AIS was delayed for Patient 1 because although the patient had syncope (a symptom of ischemic stroke) 2 days before the diagnosis of tsutsugamushi, the symptom occurred before hospitalization. Diagnosis was delayed in Patients 10 and 11 because they were not hospitalized in the neurology department, which delayed their neurological evaluation. Patient 14 developed AIS shortly after tsutsugamushi treatment and was discharged from hospital, resulting in a late diagnosis. In 18 patients, AIS was diagnosed ˂10 days after tsutsugamushi infection (Table 1).

According to a recent multi-institute registry study in South Korea, the prevalence of stroke risk factors in patients with stroke due to “other determined etiology” such as hypercoagulable state was 47.5, 23.8, 20.6, 3.2, and 21.2% for hypertension, dyslipidemia, diabetes, atrial fibrillation, and current smoking, respectively [14]. The results of our study showed that the prevalence of risk factors was 54.5, 31.8, 22.7, 22.7, 9.1, and 9.1% for hypertension, dyslipidemia, diabetes mellitus, atrial fibrillation, coronary artery disease, and current smoking, respectively; these rates were generally higher than those of patients with stroke due to “other determined etiology” (Table 2). This difference was likely due to tsutsugamushi patients with AIS having an infectious disease (tsutsugamushi). Because people with multiple concomitant diseases or poor immunity due to an underlying disease are more likely to have an infectious disease, the patients in the present study may have had more underlying diseases than those in the general population.

The incidence of fever was significantly lower in tsutsugamushi patients with AIS than in those without AIS at the time of admission (27.3 vs. 93.8%, *p* < 0.001; Table 2), which was believed to be related to the sequence of symptoms resulting from tsutsugamushi infection. Fever, one of the most common symptoms of tsutsugamushi, appears relatively early after the infection [15], whereas coagulopathy is a late manifestation of the illness [12]. Considering the sequence of these symptoms and the fact that fever is a mechanism that protects the body against infection [16], the development of fever early in the course of tsutsugamushi infection is believed to trigger the body’s defense mechanism against *O. tsutsugamushi*. However, we suspect that patients who did not develop fever early in the course of their infection did not adequately deal with the bacterial infection; instead, the disease progressed to a coagulation disorder, which is a late manifestation. This hypothesis agrees with previous reports that DIC is more common in tsutsugamushi patients with severe illness than in those with less severe illness [4]. In addition to fever, the incidence rates of headache, sore throat, dyspnea, and thirst were significantly lower in tsutsugamushi patients with AIS than in those without AIS (Table 2; Fig. 1). This finding was expected because headache, sore throat, dyspnea, and thirst are secondary symptoms of fever.

Regarding stroke risk factors, tsutsugamushi patients with AIS had significantly higher rates of hypertension, diabetes mellitus, atrial fibrillation, and dyslipidemia than those without AIS (Table 2; Fig. 1). These results suggest that tsutsugamushi infection is more likely to cause AIS in patients with stroke risk factors than in those without risk factors. Several previous studies have reported that common acute infections trigger ischemic stroke [3, 17]. However, we believe that the triggering action of these infections targets the stroke risk factors. For example, if a patient with underlying atrial fibrillation is infected with tsutsugamushi, a thrombus is more likely to develop because of an increase in heart rate in response to the infection, thereby increasing the likelihood of AIS development. This can be explained by the results of previous studies that showed the following: (1) thrombus formation was the pathogenesis of atrial fibrillation to induce AIS [18] and (2) heart rate increased during infection and the risk of incident venous thromboembolism and inflammatory and coagulation factors increased when the heart rate increased [19]. This indicates that tsutsugamushi infection acts as a trigger, further aiding the patient’s pre-existing risk factors to cause AIS. Our hypothesis needs to be confirmed in a larger systematic study.

The aPTT, D-dimer, and FDP levels were significantly higher in the target patient group than in the control group (*p* = 0.009, 0.005, and 0.009, respectively). Elevated aPTT, D-dimer, and FDP levels were associated with coagulation disorders. Therefore, the results suggest that the mechanism by which tsutsugamushi infection induces AIS is related to coagulation diseases, which agrees with the results of previous studies [4]. Furthermore, from multivariate logistic analysis, we identified D-dimer elevation as a factor influencing the development of AIS after tsutsugamushi infection (adjusted OR, 1.58; 95% CI, 1.06–2.34; *p* = 0.023). Dardiotis et al. [20] reported that the secretion of inflammatory cytokines by cancer cells triggered an inflammatory response that created a hypercoagulable state, resulting in systemic and cerebral arterial or venous thrombosis that could induce ischemic stroke. In addition, D-dimer elevation was observed in patients with cancer-related stroke, and brain imaging showed infarction of multifocal lesions [20]. Moreover, Jackson et al. [21] reported that inflammatory and hemostatic responses were activated in response to an infection. Based on the results of these previous studies and the present study, we believed that tsutsugamushi patients with a higher D-dimer level were more likely to develop AIS than those with a lower D-dimer level at the time of admission and progress to embolic stroke due to their hypercoagulable state. This study examined D-dimer levels in 19 of 22 AIS patients with tsutsugamushi infection, after excluding three patients without imaging information, and found that the D-dimer level of 16 patients exceeded the threshold of 0.5 mg/L (Supplementary table). Among these 16 patients, 13 showed embolic patterns on brain MRI, thereby supporting our hypothesis (Supplementary table). However, we could not determine whether higher D-dimer levels induced embolic stroke. Because atrial fibrillation, another risk factor, can cause a cardioembolic stroke by abnormally promoting thromboembolism [22], the five patients with atrial fibrillation, elevated D-dimer level (≥ 0.5 mg/dL), and an embolic pattern lesions on DWI could have developed cardioembolic stroke due to atrial fibrillation. However, the eight patients who showed embolic-pattern lesions with D-dimer elevation but without atrial fibrillation could serve as evidence to support our proposed hypothesis. Although a previous study reported that tsutsugamushi infection can elevate coagulation factors, such as D-dimer, and cause DIC to induce bleeding [4], its association with ischemic stroke has not yet been reported. Additionally, previous studies have hypothesized that tsutsugamushi infection-induced coagulopathy affects the onset of stroke [12, 13]; however, all of these studies were case reports. The novelty of this study lies in its evaluation of the association between tsutsugamushi infection and AIS by analyzing brain images and data. The novelty of this study lies in its evaluation of the association between tsutsugamushi infection and AIS by analyzing brain images and data. We figured out what pattern of stroke image the tsutsugamushi patients with AIS have and also grasped which risk factors and lab findings they commonly have by retrospectively evaluating multiple patients.

This study had several limitations. First, we could not control all confounding variables because this was a retrospective study. Therefore, this study could not regulate variables other than tsutsugamushi infection that could cause AIS. Future prospective cohort studies are required to improve the reliability of these results. Second, because the data were collected at a tertiary hospital, many patients had severe diseases, which could have introduced selection bias. However, because most people in South Korea visit tertiary hospitals when they suspect a stroke, it is difficult to find cases of tsutsugamushi co-occurring with AIS in small primary care clinics. Therefore, we believe that the results will be similar, even after including patients from primary and secondary hospitals. Third, it is necessary to exclude the possible influence of atrial fibrillation history on the stroke pattern to explain the correlation between D-dimer and embolic patterns in the stroke pattern of tsutsugamushi patients. However, this study could not compare patients with atrial fibrillation history and those without atrial fibrillation history. Therefore, it is imperative to conduct histological studies to determine whether excessive thrombin production due to hypercoagulable state, rather than atherosclerosis, causes embolic stroke. However, histological samples could not be obtained from stroke patients. Finally, the sample size was small because the co-occurrence of tsutsugamushi and AIS is rare. There may have been statistical bias because of the small sample size. For the same reason, we could not perform statistical analysis to determine whether the brain MRI embolic pattern in the target group was associated with D-dimer levels. However, it was meaningful to collect data from 22 patients because no other study has collected data from as many tsutsugamushi patients with AIS as this study did.

## Conclusions

AIS in patients with tsutsugamushi tended to appear in an embolic pattern rather than in a non-embolic pattern on brain MRI and occurred more easily in patients with stroke risk factors. Moreover, these patients were less likely to have fever and had higher D-dimer levels. We believe that D-dimer levels play an important role in the pathophysiology of tsutsugamushi infection and increase the incidence of AIS. We believe that tsutsugamushi infection increased the likelihood of developing AIS in individuals with stroke risk factors. We suspect that if a patient does not develop fever early in the course of tsutsugamushi infection, the patient may develop AIS after a few days. In conclusion, we believe that if a patient with tsutsugamushi presents with elevated D-dimer levels or no fever upon admission, AIS should be considered.

### Electronic supplementary material

Below is the link to the electronic supplementary material.

Supplementary Material 1

## Data Availability

The data supporting the findings of this study are available from the corresponding author upon reasonable request.

## Abbreviations

AIS: Acute ischemic stroke
TIA: transient ischemic stroke
DIC: disseminated intravascular coagulation
PCR: polymerase chain reaction
MRI: magnetic resonance imaging
AST: spartate aminotransferase
ALT: alanine aminotransferase
WBC: white blood cell
Hg: hemoglobin
PLT: platelet
PT: prothrombin time
aPTT: activated partial thromboplastin time
FDP: fibrin degradation product
DWI: diffusion-weighted imaging
