# Supplementary material for: Acute ischemic stroke in Tsutsugamushi: understanding the underlying mechanisms and risk factors
Source: BMC Neurol. 2024 Jan 24;24:42. doi: 10.1186/s12883-024-03534-1 (PMC10807141; doi:10.1186/s12883-024-03534-1)
Supplement: Supplementary file 1 — Supplementary Material 1 [file 12883_2024_3534_MOESM1_ESM.docx]

Supplementary table. Diffusion-weighted imaging of a patient with acute ischemic stroke, with tsutsugamushi and stroke patterns (embolic, non-embolic) and risk factors of the patients (Normal range of D-dimer value <0.5mg/l)

| Patient | MR image | Lesion pattern | Atrial fibrillation | D-dimer level (mg/L) |
| --- | --- | --- | --- | --- |
| 1 | 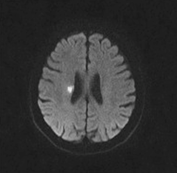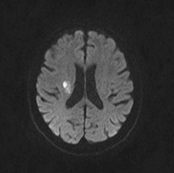 | non-embolic | - | 0.279 |
| 2 | 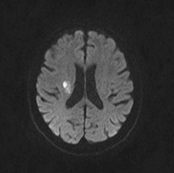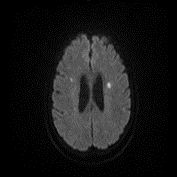 | embolic | + | 5.003 |
| 3 | 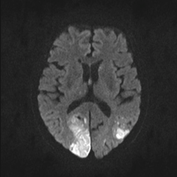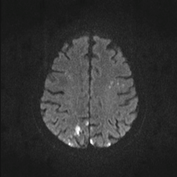 | embolic | - | 257.136 |
| 4 | 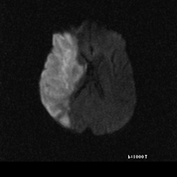 | embolic | + | 1.050 |
| 5 | 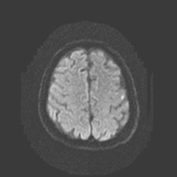 | embolic | - | 1.570 |
| 6 | 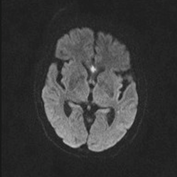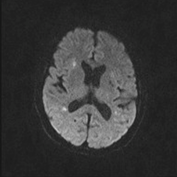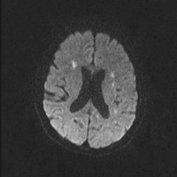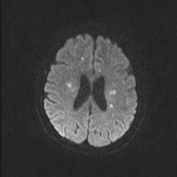 | embolic | + | 1.152 |
| 7 | 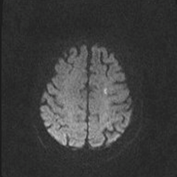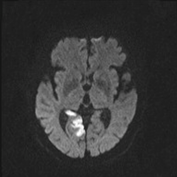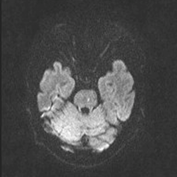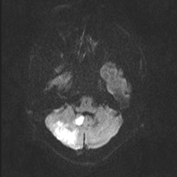 | embolic | - | 0.438 |
| 8 | 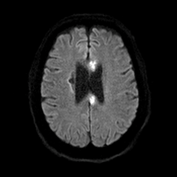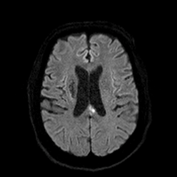 | embolic | - | 2.057 |
| 9 | 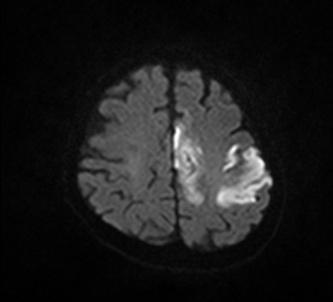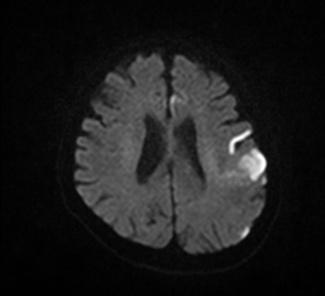  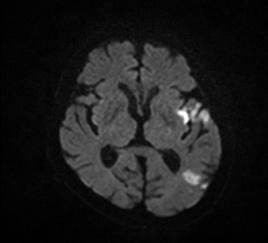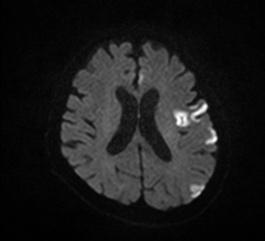 | embolic | + | 0.210 |

| 10 | 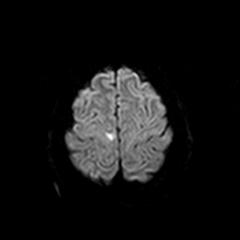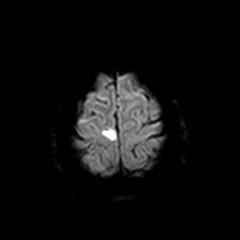 | non-embolic | - | 34.32 |
| --- | --- | --- | --- | --- |
| 11 | 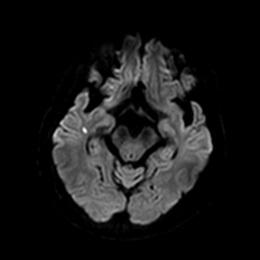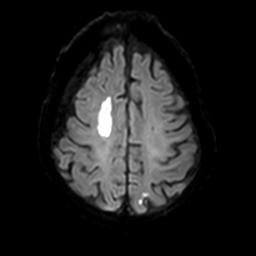 | embolic | - | 1.4 |
| 12 | 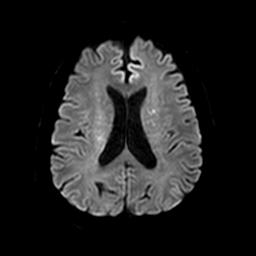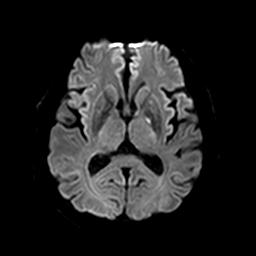 | embolic | - | 18.96 |
| 14 | 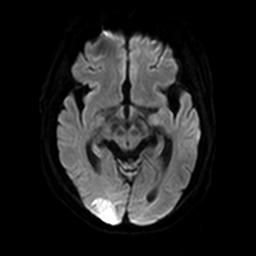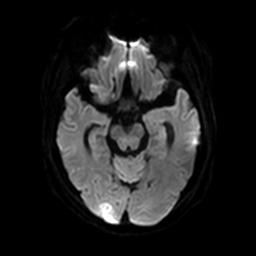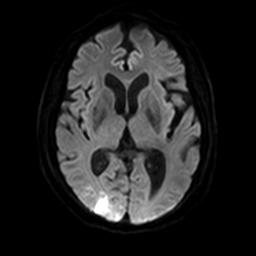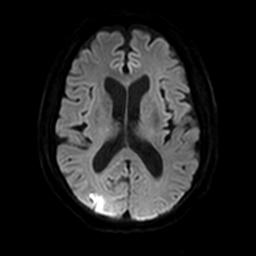 | embolic | - | 6.27 |
| 15 | 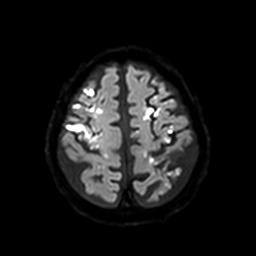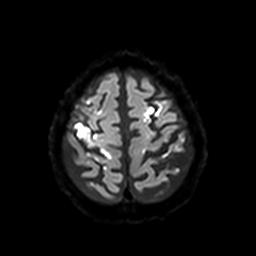  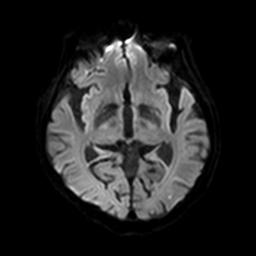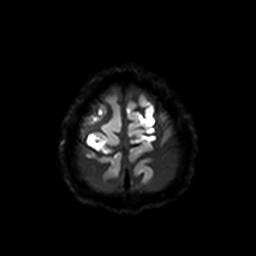 | embolic | - | 5 |
| 16 | 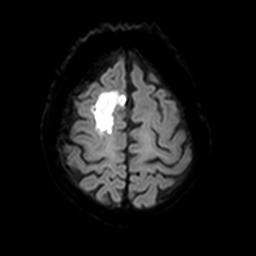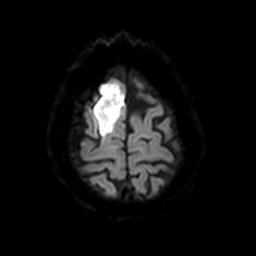 | non-embolic | - | 9.48 |
| 17 | 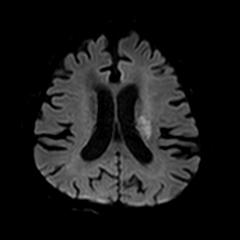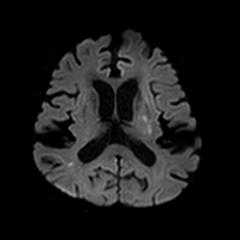 | embolic | + | 5.83 |

| 19 | 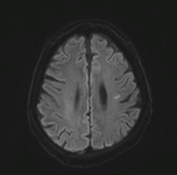 | non-embolic | | - | 5.74 |
| --- | --- | --- | --- | --- | --- |
| 21 | 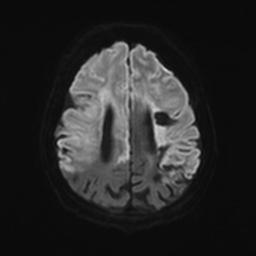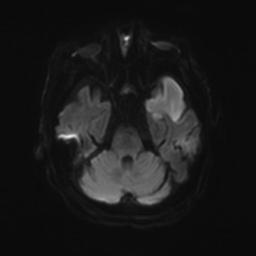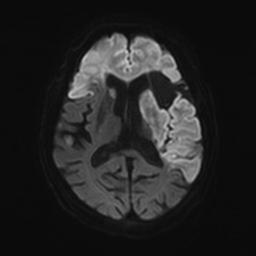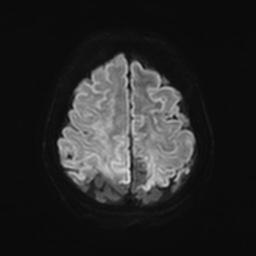 | | embolic | + | 2.67 |
| 22 | 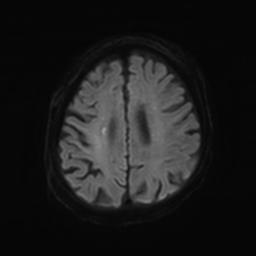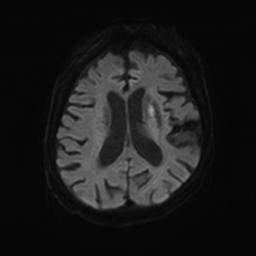 | | embolic | - | 17.47 |
